# Rural-urban disparities and trends in cancer screening: an analysis of Behavioral Risk Factor Surveillance System data (2018-2022)

**DOI:** 10.1093/jncics/pkae113

**Published:** 2024-11-09

**Authors:** Gabriel A Benavidez, Ami E Sedani, Tisha M Felder, Matthew Asare, Charles R Rogers

**Affiliations:** Department of Public Health, Robbins College of Health and Human Sciences, Baylor University, Waco, TX 76798, United States; Men’s Health Inequities Research Lab, Milwaukee, WI 53226, United States; Department of Biobehavioral Health & Nursing Science, Arnold School of Public Health, University of South Carolina, Columbia, SC 29208, United States; Department of Public Health, Robbins College of Health and Human Sciences, Baylor University, Waco, TX 76798, United States; Men’s Health Inequities Research Lab, Milwaukee, WI 53226, United States

## Abstract

**Background:**

Despite evidence of the benefit of routine cancer screenings, data show a concerning decline in cancer screening uptake for multiple cancers. This analysis aimed to examine rural-urban differences in recent trends for being up-to-date with screenings for breast, cervical, and colorectal cancers.

**Methods:**

We used 2018, 2020, and 2022 Behavioral Risk Factor Surveillance System data to assess up-to-date cancer screening status among eligible adults in the United States. We calculated weighted prevalence estimates overall and stratified by county-level rural-urban classification. We used survey-weighted multivariable logistic regression models to examine rural-urban disparities in cancer screening up-to-date status by year.

**Results:**

Prevalence of being up-to-date with each cancer screening was lower in 2022 than it was in 2018. The largest decline in screening overall was for cervical cancer, which dropped from 81.89% in 2018 to 47.71% in 2022. Rural-urban disparities were observed for breast cancer screening from 2018 to 2022, with the odds of up-to-date screening being 14% to 27% lower for rural populations than for urban populations. For colorectal and cervical cancers, the odds of being up-to-date with screenings were lower for rural populations in 2018 and 2020, but no statistically significant difference was observed in 2022 (colorectal screening odds ratio = 0.96, 95% CI = 0.90 to 1.02; cervical screening odds ratio = 0.97, 95% CI = 0.93 to 1.03).

**Conclusion:**

There is a concerning trend of decreasing uptake of cancer screenings that will challenge future efforts in cancer prevention and control. There is a need to better understand the factors contributing to the decline in cancer screening update.

## Introduction

In the United States, colorectal, breast, and cervical cancers together account for more than 40% of cancer incidence and nearly 25% of all cancer deaths among women.^1^ For men, colorectal cancer (CRC) alone represents nearly 10% of total cancer incidence and mortality.^1^ Through routine screenings, each of these cancers is amenable to early detection, with survival rates at or above 90% when detected at a localized stage.^2^ Over the past 3 decades, substantial progress in cancer control has led to a consistent decline in overall cancer mortality, from 214 deaths per 100 000 in 1990 to 143 deaths per 100 000 in 2020,^3^ much of which can be attributed to successful public health efforts to increase early detection and treatment of these cancers through screening. Model-based estimates suggest a 10% increase in the proportion of age-eligible people who complete routine cancer screenings would save an additional 1300 lives from breast cancer, 3400 lives from cervical cancer, and 11 000 lives from CRC,^4^ demonstrating the importance of routine cancer screenings in further improving cancer control efforts.

Recent data show stagnant and even declining prevalence of eligible individuals up-to-date with current screening guidelines for breast, cervical, and colorectal cancers. In a study examining national cancer screening rates from 2000 to 2015, Hall et al.^5^ observed a statistically significant decline in the proportion of eligible women receiving both Papanicolaou tests and mammograms. The study also revealed that only CRC screening rates had increased over time, raising particular concerns for socially disadvantaged groups—such as individuals of low socioeconomic status, racial and ethnic groups underserved in medicine, and residents of rural areas. Previous research has consistently highlighted disparities in up-to-date screening among these groups, which may be increasing over time.^6^

Of special interest are rural populations, which face unique challenges in achieving positive cancer health outcomes. For example, studies have shown that the incidence of breast cancer is higher in urban areas than in rural areas,^7^ yet when examined by stage at diagnosis, rural women have a higher proportion of cancers diagnosed at more distant stages and have worse survival outcomes.^8^^,^^9^ In 2018, the American Society of Clinical Oncology launched a rural cancer care initiative to better understand the factors influencing these disparities; it identified that rural areas face the combined adverse effects of low socioeconomic status and a lack of physical access to oncology-related health-care services (preventive and therapeutic).^10^

Because of documented disparities in cancer outcomes between rural and urban populations, coupled with a national decline in cancer screening rates, there is a critical need to investigate current trends in cancer screening across rural and urban areas, a gap that remains unaddressed. Examining and comparing rural-urban cancer screening trends over time in the United States offers the opportunity to monitor advancement toward Healthy People 2030 national goals and progress toward eradicating rural-urban inequities in cancer.^11^ Understanding current trends is vital, especially considering the major disruptions caused by the COVID-19 pandemic in 2020, which led millions of individuals to skip routine screening. The world is still grappling with the unknown long-term effects of that period. Using the latest data from the Behavioral Risk Factor Surveillance System (BRFSS; 2018, 2020, and 2022), this study aimed to (1) assess guideline adherence rates for breast, colorectal, and cervical cancer screening based on rural-urban status; (2) analyze trends in up-to-date screening among rural and urban populations; and (3) examine potential changes in screening prevalence across rural and urban areas over time, assessing whether screening disparities have worsened.

## Methods

### Data source

This repeated cross-sectional study used freely accessible, deidentified data from the 2018, 2020, and 2022 survey cycles of the BRFSS, with respective median responses rates of 48.40%, 47.9%, and 45.0%.^12^ The BRFSS is the largest annual nationally representative telephone survey conducted among noninstitutionalized adults (ie, individuals ≥18 years of age) in the United States on health behavior, health care, and health status across all US states and territories.^13^ The questionnaire consists of a core component (the fixed core, rotating core, and emerging core), optional modules, and state-added questions.^13^ Cancer screening questions are part of the rotating core component asked by all states in even years. Additional details on BRFSS methods are publicly available.^13^ The study had no direct involvement of the human participants; therefore, ethical approval was not sought. All data used for this study are free to use and publicly available (https://www.cdc.gov/brfss/annual_data/annual_data.htm).

### Study sample

We developed separate cohorts for each cancer screening test (cervical, breast, and colorectal) for each year of BRFSS data. Respondents from Guam and Puerto Rico were excluded from the analysis. Our analysis was restricted to individuals who were age eligible based on the best evidence of benefit according to the US Preventive Services Task Force (USPSTF) recommendations for each cancer screening type.^14^ For breast cancer age eligibility, best available evidence suggests that women between the ages of 50 and 74 years should be screened. For CRC, the best available evidence suggests that persons between the ages of 50 and 75 be screened. Although the USPSTF does suggest evidence of a benefit to screening for CRC starting at 45 years of age, the BRFSS did not begin asking persons younger than 50 years about their CRC screening history before 2022. Therefore, we restricted our sample to individuals aged 50 to 75 years for CRC screening in each year of the BRFSS. For cervical cancer, the best available evidence suggests that women between 21 to 65 years of age be screened. Women who had a hysterectomy were excluded from cervical screening eligibility. Female gender was determined based on BRFSS survey respondents classifying their sex at birth as female. SAS code used to generate the study sample can be found in the supplementary material online.

### Measures

The primary outcome for this study was whether an eligible individual was self-reported as being up-to-date with cancer screening (ie, guideline adherent). For this analysis, respondents were considered up-to-date with screening if they met the USPSTF recommendations for screening modality and frequency.^14^ For breast cancer screening, age-eligible women were considered up-to-date if they had received a mammogram within the past 2 years. For CRC screening, age-eligible respondents were considered up-to-date if they met any of the following criteria: had received a colonoscopy within the past 10 years, flexible sigmoidoscopy within the past 5 years, or stool-based test within the past year. For cervical cancer screening, age-eligible women were considered up-to-date if they had received a Papanicolaou test (cervical cytology) within the past 3 years or, if they were 30 to 65 years of age, a high-risk human papillomavirus test within the past 5 years.

The independent variable of interest was county-level rurality of the respondent’s county of residence (rural, urban). In 2018, BRFSS introduced a county-level urban-rural variable based on the 2013 National Center for Health Statistics Rural-Urban classification scheme, where urban counties received values of 1 to 5 and rural counties were assigned a value of 6.^15^ Other covariates examined include self-reported gender (male, female; defined as sex assigned at birth), age, race and ethnicity (non-Hispanic White, non-Hispanic Black, non-Hispanic Asian, non-Hispanic American Indian or Alaska Native, Hispanic, non-Hispanic Other), educational attainment (less than a high school diploma or General Educational Development test, high school diploma/General Educational Development test, some college or technical school, college or technical school graduate), health insurance status (yes, no), and medical cost as a barrier to receiving health care in the past 12 months (yes, no).

### Statistical analysis

Accounting for the complex survey sampling methodology of the BRFSS, we produced weighted prevalence estimates and 95% CIs of up-to-date screening status for breast, cervical, and colorectal cancer screening by year and stratified by rural and urban county of residence using the BRFSS generated survey weights. We then used Rao-Scott χ^2^ tests^16^ to compare differences in up-to-date screening prevalence for breast, cervical, and colorectal cancer screening across rural-urban status.

To examine the trend in up-to-date screening status over time, we used a weighted logistic regression model^17^ using the *PROC SURVEYLOGISTIC* procedure in SAS, version 9.4, statistical software (SAS Institute Inc), with up-to-date cancer screening status as the dependent variable and BRFSS survey year as the independent variable. In this model, we modeled the log-odds of a person being up-to-date for the cancer screening of interest. Using multivariable logistic regression, we examined the association between rural-urban residence and the odds of being up-to-date with cancer screenings, adjusting for age, gender (only among CRC cancer screening), insurance status, race and ethnicity, educational attainment, and medical cost as a barrier to receiving health care. A high proportion of participants had missing data for the variable income (>10% for each screening type and survey year); thus, income was not included in the following modeling procedures. For all logistic regression models, each cancer type was examined individually and was modeled in an overall analysis and additionally stratified by rural-urban county of residence. A 2-tailed ɑ = .05 was considered statistically significant for all hypothesis tests.

## Results

### Respondent characteristics

Study population characteristics are shown in Table 1 for each screening-eligible cohort by year and cancer screening type. There were unweighted totals of 302 968 (2018 = 108 746; 2020 = 93 531; 2022 = 100 691) eligible respondents for breast cancer screening, 313 035 (2018 = 108 777; 2020 = 98 352; 2022 = 105 906) eligible respondents for cervical cancer screening, and 581 477 (2018 = 198 862; 2020 = 172 706; 2022 = 209 909) eligible respondents for CRC screening among the 3 cycles of BRFSS data. Across each cancer screening type and year, the highest proportion of eligible participants self-reported being non-Hispanic White, making more than $75 000 per year, being a college graduate, having some form of health insurance, and living in an urban county.

**Table 1.** Demographics of the screening-eligible population, by year and cancer screening type

	2018			2020			2022		
	**Breast cancer (n = 108** **746)^a^**	**Cervical cancer (n = 10** **877)^a^**	**Colorectal cancer (n = 198** **862)^a^**	**Breast cancer (n = 93** **531)^a^**	Cervical cancer (n = 98, 352)^a^	Colorectal cancer (n = 172 706)^a^	**Breast cancer (n = 100** **691)^a^**	**Cervical cancer (n = 105** **906)^a^**	**Colorectal cancer (n = 209** **909)^a^**
Age, median (IQR), y	63.00 (57.0-68.00)	47.00 (35.00-57.00)	63.00 (57.00-69.00)	63.00 (57.00-69.00)	46.00 (35.00-56.00)	63.00 (57.00-69.00)	63.00 (57.00-69.00)	46.00 (35.00-57.00)	62.00 (54.00-69.00)
Sex, %									
Male	—	—	44.72	—	—	44.97	—	—	46.46
Female	—	—	55.28	—	—	55.03	—	—	53.54
Race and ethnicity, %									
Non-hispanic American Indian or Alaska Native	1.91	2.18	1.87	1.7	2.02	1.66	1.61	1.88	1.67
Hispanic	4.54	9.79	4.51	6.26	12.11	6.06	5.5	11.4	6.28
Non-Hispanic Asian	1.34	2.62	1.49	1.55	3.14	1.62	1.56	3.53	1.95
Non-Hispanic Black	8.99	9.63	7.93	7.98	8.57	7.19	8.91	9.23	8.36
Non-Hispanic White	80.82	72.52	81.59	79.75	70.16	80.61	80.51	70.87	79.67
Other	2.40	3.26	2.6	2.75	4.00	2.86	1.91	3.09	2.06
Income, %									
<$25 000	26.2	24.11	23.81	24.31	22.89	22.3	17.62	14.89	15.72
$25 000-$50 000	24.2	21.39	23.36	23.89	21.07	22.59	25.39	22.47	23.29
$51 000-75 000	17.22	15.99	17.12	17.17	15.93	16.95	17.91	16.19	17.05
>$75 000	32.37	38.52	35.71	34.63	40.11	38.16	39.08	46.46	43.94
Education, %									
Less than high school	6.11	6.05	6.46	5.49	5.45	5.85	4.75	5.00	5.49
High school graduate	25.95	21.59	26.54	24.57	21.02	25.47	21.97	18.88	23.13
Some college	29.30	27.75	27.75	29.67	27.95	28.31	29.67	26.62	27.75
College graduate	38.64	44.61	39.24	40.28	45.58	40.37	43.61	49.50	43.62
Insurance status, %									
Some form of health insurance	95.28	89.74	94.66	95.04	89.40	94.48	96.01	91.78	94.83
No form of health insurance	4.72	10.26	5.34	4.96	10.60	5.52	3.99	8.22	5.17
Cost is a barrier to receive medical care, %									
No	90.45	85.77	91.44	92.45	87.80	93.14	93.04	87.67	93.02
Yes	9.55	14.23	8.56	7.55	12.20	6.86	6.96	12.33	6.98
Rurality, %									
Rural	17.01	13.95	16.89	16.83	13.14	16.74	14.33	10.93	13.83
Urban	82.99	86.05	83.11	83.17	86.86	83.26	85.67	89.07	86.17

a Unweighted number of Behavioral Risk Factor Surveillance System respondents eligible to receive screening based on US Preventive Services Task Force criteria.

### Breast cancer

**Figure 1.**
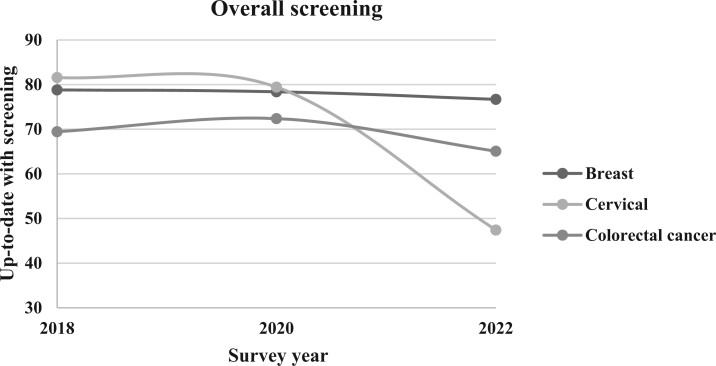
Overall prevalence of up-to-date cancer screenings, 2018-2022. This line graph illustrates the survey-weighted percentage of respondents up-to-date with screenings for breast, cervical, and colorectal cancers according to Behavioral Risk Factor Surveillance System survey years 2018, 2020, and 2022.

**Figure 2.**
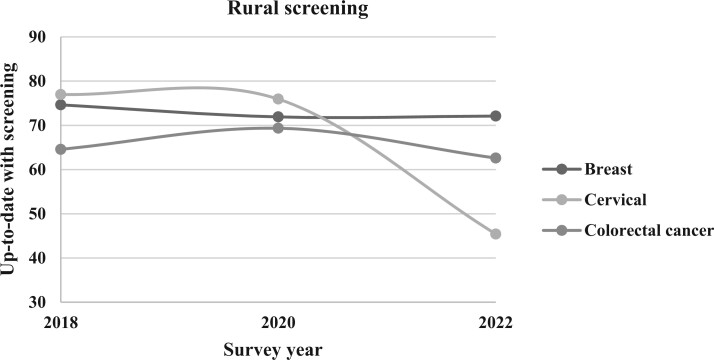
Rural prevalence of up-to-date cancer screening, 2018-2022. This line graph illustrates the survey-weighted percentage of respondents from rural counties up-to-date with screenings for breast, cervical, and colorectal cancers according to Behavioral Risk Factor Surveillance System survey years 2018, 2020, and 2022. Rurality in this study was defined using the Behavioral Risk Factor Surveillance System county-level urban-rural variable, based on the 2013 National Center for Health Statistics Rural-Urban classification.

**Figure 3.**
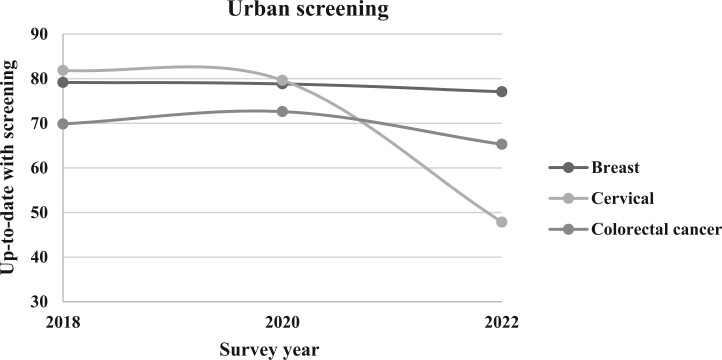
Urban prevalence of up-to-date cancer screening, 2018-2022. This line graph illustrates the survey-weighted percentage of respondents from urban counties up-to-date with screenings for breast, cervical, and colorectal cancers according to Behavioral Risk Factor Surveillance System survey years 2018, 2020, and 2022. Rurality in this study was defined using the Behavioral Risk Factor Surveillance System county-level urban-rural variable, based on the 2013 National Center for Health Statistics Rural-Urban classification.

Between 2018 and 2022, the estimated overall weighted prevalence of women aged 50 to 74 years who were up-to-date with breast cancer screening declined from 78.82% to 76.70% (Table 2; Figure 1). Among women residing in rural counties (Figure 2), in 2018 74.62% were up-to-date with screening compared with 79.15% of women in urban counties (Figure 3). There remained a statistically significant difference in the prevalence of women up-to-date with breast cancer screening between urban and rural women in 2020 (78.85% vs 71.9%; *P* < .001) and in 2022 (77.06% vs 72.1%; *P* < .001), but among women from both rural and urban counties, the prevalence of women up-to-date with breast cancer screening had declined since 2018. Overall, compared with eligible women in 2018, eligible women in 2020 (odds ratio [OR] = 0.95, 95% CI = 0.93 to 0.97) and 2022 (OR = 0.93, 95% CI = 0.91 to 0.95) had statistically significant lower odds of being up-to-date with breast cancer screening. This same trend was observed when stratified by rural-urban status, except for in 2020 among urban women, where the odds of being up-to-date were not different than in 2018 (Table 2).

**Table 2.** Weighted prevalence estimates and odds of being up-to-date with cancer screenings, by rural-urban status and year (2018-2022)

Cancer screening type	Year	Prevalence overall (95% CI)	Prevalence rural (95% CI)	Prevalence urban (95% CI)	Overall OR (95% CI)	Rural OR (95% CI)	Urban OR (95% CI)
Breast cancer	2018^a^	78.80 (78.22 to 79.40)	74.62 (73.21 to 76.02)	79.15 (78.52 to 79.77)	(Referent)	(Referent)	(Referent)
	2020^a^	78.40 (77.73 to 79.10)	71.90 (70.31 to 73.51)	78.85 (78.13 to 79.60)	0.95 (0.93 to 0.97)	0.87 (0.78 to 0.97)	0.98 (0.93 to 1.04)
	2022^a^	76.70 (76.10 to 77.30)	72.10 (70.35 to 73.86)	77.06 (76.43 to 77.70)	0.93 (0.91 to 0.95)	0.92 (0.87 to 0.97)	0.86 (0.84 to 0.93)
Cervical cancer	2018^a^	81.59 (81.09 to 82.10)	76.97 (75.55 to 78.40)	81.85 (81.34 to 82.40)	(Referent)	(Referent)	(Referent)
	2020^a^	79.44 (78.86 to 80.00)	75.94 (74.29 to 77.58)	79.64 (79.05 to 80.25)	0.87 (0.83 to 0.91)	0.94 (0.84 to 1.06)	0.94 (0.83 to 0.91)
	2022	47.71 (47.10 to 48.33)	45.40 (43.28 to 47.53)	47.85 (47.21 to 48.49)	0.21 (0.02 to 0.22)	0.20 (0.19 to 0.21)	0.25 (0.22 to 0.28)
Colorectal cancer	2018^a^	69.45 (68.90 to 69.90)	64.57 (63.36 to 65.79)	69.85 (69.34 to 70.40)	(Referent)	(Referent)	(Referent)
	2020^a^	72.38 (71.80 to 72.90)	69.34 (68.18 to 70.50)	72.62 (72.03 to 73.20)	1.15 (1.11 to 1.19)	1.18 (1.14 to 1.23)	1.24 (1.15 to 1.34)
	2022^a^	65.08 (64.60 to 65.60)	62.59 (61.34 to 63.84)	65.28 (64.78 to 65.79)	0.64 (0.63 to 0.67)	0.79 (0.76 to 0.82)	0.75 (0.70 to 0.81)

Abbreviation: OR = odds ratio.

a Statistically significant difference based on Rao-Scott χ^2^ tests, with ɑ = .05.

Although declines in screening prevalence were observed for both rural and urban women over time, there remained a notable disparity between rural and urban women at each time point. The adjusted odds of a women from a rural county being up-to-date with breast cancer screening was 0.85 (95% CI = 0.78 to 0.94) times that of a woman from an urban county in 2018 (OR = 0.73, 95% CI = 0.66 to 0.81, in 2020; OR = 0.86, 95% CI = 0.77 to 0.96, in 2022) (Table 3).

**Table 3.** Adjusted odds of being up-to-date with screenings, by rural county classification^a^

	Breast cancer screening		Colorectal cancer screening		Cervical cancer screening	
Year	Rural adjusted OR (95% CI)	Urban	Rural adjusted OR (95% CI)	Urban	Rural adjusted OR (95% CI)	Urban
2018	0.85 (0.78 to 0.94)	(Referent)	0.84 (0.78 to 0.89)	(Referent)	0.77 (0.74 to 0.81)	(Referent)
2020	0.73 (0.66 to 0.81)	(Referent)	0.89 (0.84 to 0.96)	(Referent)	0.78 (0.73 to 0.81)	(Referent)
2022	0.86 (0.77 to 0.96)	(Referent)	0.96 (0.90 to 1.02)	(Referent)	0.97 (0.93 to 1.03)	(Referent)

Abbreviation: OR = odds ratio.

a Adjusted for age, sex (colorectal cancer), educational status, insurance status, medical cost as a barrier, and race.

### Colorectal cancer

In 2018, approximately 69.45% of age-eligible people were up-to-date with USPSTF CRC screening guidelines (Figure 1; Table 2). By 2020 the estimated proportion of people up-to-date with CRC screening increased to 72.38%; however, in 2022 only 65.08% of eligible individuals were up-to-date with CRC screening. Similar patterns were observed when examined by county-level rurality (Figures 2 and 3). A higher proportion of urban residents were up-to-date with CRC screening than rural residents in 2018 (69.85% vs 64.57%), 2020 (72.62% vs 69.34%), and 2022 (65.28% vs 62.59%).

When comparing each year with 2018, the odds of being up-to-date for CRC screening was 1.15 times higher overall in 2020 (95% CI = 1.11 to 1.19), 1.18 times higher among rural residents (95% CI = 1.14 to 1.23), and 1.24 times higher among urban residents (95% CI = 1.15 to 1.34) (Table 2). In contrast, in 2022 compared with 2018, the odds of being up-to-date with CRC screening was lower overall (OR = 0.64, 95% CI = 0.63 to 0.67) and for both rural (OR = 0.79, 95% CI = 0.76 to 0.82) and urban (OR = 0.75, 95% CI = 0.70 to 0.81) respondents.

In adjusted analyses, there were statistically significant rural-urban disparities in up-to-date screening in 2018 and in 2020 (Table 3). In 2018, compared with an urban respondent, a rural respondent had 0.84 times the odds of being up-to-date with CRC screening. Again in 2020, a rural respondent had 0.90 times the odds of being up-to-date with CRC screening compared with an urban respondent. In 2022, there was no statistically significant difference in the odds (OR = 0.96, 95% CI = 0.90 to 1.02) of being up-to-date with CRC screening between rural and urban residents.

### Cervical cancer

In 2018, the estimated overall prevalence of eligible women up-to-date with cervical cancer screening was 81.59% nationally, 76.97% in rural counties, and 81.85% in urban counties (Table 2; Figure 1). By 2020, the overall weighted national prevalence decreased to 79.44% and to 47.71% in 2022. In rural counties, the prevalence of up-to-date screening (Table 2; Figure 2) decreased to 75.94% in 2020 to 45.40% in 2022. In urban counties, the prevalence of up-to-date screening (Table 2; Figure 3) decreased to 79.64% in 2020 and to 47.85% in 2022.

Compared with 2018, the odds of being up-to-date with cervical cancer screening decreased overall and among rural and urban counties in each cycle of the BRFSS examined. Compared with 2018, the odds of being up-to-date with cervical cancer screening in 2020 was 0.87 (95% CI = 0.84 to 0.88) and in 2022 was 0.21 (95% CI = 0.20 to 0.22). In addition, over time the disparities in up-to-date screening between rural and urban respondents narrowed. In 2018, compared with women in urban counties, women in rural counties had 0.77 times (95% CI = 0.74 to 0.81) the odds of being up-to-date with cervical cancer screening. This rate remained consistent in 2020: The odds of a woman in a rural county being up-to-date with cervical cancer screening was 0.78 (95% CI = 0.73 to 0.81) times that of a woman in an urban county. By 2022, there was no statistically significant difference in the odds of being up-to-date with cervical cancer screening between rural and urban women (OR = 0.97, 95% CI = 0.93 to 1.03).

## Discussion

This analysis examined recent rural and urban trends in up-to-date screening status for breast, colorectal, and cervical cancers from 2018 to 2022. Despite the well-documented benefits of regular cancer screening to increase early detection and reduce cancer mortality, our findings demonstrate a concerning trend of stagnation and even decline in the prevalence of up-to-date screening status for breast, colorectal, and cervical cancers in recent years among rural and urban populations. For each cancer, compared with 2018, the odds of being up-to-date with screening was lower overall among rural and urban respondents alike. The most noticeable decline in up-to-date screening was for cervical cancer screening, with an approximately 30.0% decline in the prevalence of up-to-date screening overall and across rural and urban respondents. Notably, the disparities in up-to-date screening status for cervical and colorectal cancers among rural respondents compared with urban respondents in 2018 was no longer observed in 2022, suggesting that although screening prevalence has been declining, the disparities for rural respondents have not widened as would have been expected.

Studies have demonstrated that the onset of the COVID-19 pandemic led to missed health-care visits,^18^ resulting in declines in cancer screenings in the subsequent year.^19^^,^^20^ In a study examining data from the US Health Care Cost Institute on changes in the number of cancer screenings performed from 2019 to 2020, Concepcion and colleagues (2023)^20^ reported a 25% decrease in the number of colonoscopies performed and a 16% decrease in the number of mammograms performed. Yet, cancer screenings were already declining before the COVID-19 pandemic. Hall et al.^5^ found that between 2000 and 2015, the proportion of age-eligible individuals up-to-date with their screenings had declined over time, with only CRC screening demonstrating an increase. Using data from the National Health Interview Survey, Suk and colleagues^21^ also found that the proportion of age-eligible women who were not up-to-date with cervical cancer screening had increased nearly 10% from 2005 to 2019 before the start of the pandemic. It is difficult to discern what proportion of the decreased prevalence of up-to-date screening among rural and urban residents in our findings is the continuation of a downward trend in cancer screening or the remaining effect of the COVID-19 pandemic–related delays in seeking routine health care.

In addition, amidst the COVID-19 pandemic, political divisions regarding belief and trust in science and medicine emerged.^22^^,^^23^ If the declining trend persists after the pandemic, it may also be influenced by specific conspiracy beliefs surrounding COVID-19, potentially affecting trust in public health recommendations.^24^^,^^25^ To progress toward mitigating the impact of COVID-19–related delays on cancer screening rates and fostering a culture of trust and cooperation in health-care decision making, targeted campaigns must be implemented that raise awareness about the importance of cancer screening and address misconceptions surrounding COVID-19–related delays must be implemented. Providing accessible and accurate information can help rebuild trust in health-care systems and encourage individuals to prioritize screening.^25^^,^^26^ Moreover, an investment in robust public health systems capable of responding effectively to crises and providing ongoing support for preventive care initiatives is warranted. This investment includes building partnerships with community organizations, implementing screening reminders, and offering incentives for participation.^26^^,^^27^

Our findings demonstrate a narrowing gap in the disparity of up-to-date screening among rural and urban residents. For example, in 2022 our findings demonstrated no statistically significant difference in the odds of up-to-date screening status for CRC and cervical cancer for rural respondents compared with urban respondents. Unfortunately, this narrowing gap may be the result of a more drastic decline in up-to-date screening among urban residents rather than meaningful increases in screening among rural residents. Although efforts are needed to increase up-to-date cancer screenings to prepandemic levels, these efforts must account for rural populations and the already-limited oncology workforce and infrastructure in these areas.^28^

In 2020, our results demonstrated a noticeable increase in the proportion of eligible persons up-to-date with CRC screening overall and across rural and urban populations. It is possible that this increase was the result of increased use of noninvasive stool-based tests, such as fecal immunochemical tests (FITs). Previous reports of CRC screening during the pandemic found that although the use of colonoscopies decreased, there was an increase in the completion of FIT-DNA tests, such as Cologuard, and FIT between 2019 and 2021.^29^ Although not as effective as more invasive endoscopic procedures, FIT use may be the most sustainable way to ensure consistent CRC screening among rural residents, and their heavy use during the pandemic should be noted as proof of their utility. It is imperative to implement future interventions aimed at making FIT testing kits widely available in rural communities, including distribution through health-care professionals, pharmacies, and community health centers, because such efforts highlight the necessity of adjusting health-care delivery to cater to the diverse needs of communities, especially during difficult periods, such as the COVID-19 pandemic.^30^

This study is the first large, nationally focused effort to examine recent trends in up-to-date cancer screening status across rural and urban populations, but this work is not without limitations. The BRFSS relies on self-reported screening status, which some work has shown can be an overestimate of screening status compared with screening identification from patient medical records.^31^ Although obtaining screening status from electronic health records would likely be more representative of true screening status, self-reported data are the most efficient for national surveillance efforts, and BRFSS estimates are similar to those found in other large national health surveys.^32^^,^^33^ Our criteria for determining screening eligibility and up-to-date status are based on the highest graded evidence from the USPSTF recommendations, which may differ from recommendations from the American Cancer Society or American College of Obstetricians and Gynecologists in terms of starting age and frequency. Some have suggested that USPSTF recommendations may be too conservative or restrictive^34^; therefore, our analysis may be more conservative than if we would have used other screening criteria. That said, the BRFSS cancer screening modules and questions are designed to fall in line with USPSTF parameters.

Despite ongoing efforts, the proportion of individuals undergoing screening for breast, colorectal, and cervical cancers continues to decline. The challenges posed by the COVID-19 pandemic likely intensified this trend across urban and rural areas. Although rural-urban disparities persist, particularly in breast cancer screening, our findings suggest that these disparities have not worsened from 2018 to 2022. Nevertheless, efforts are required to restore prepandemic screening levels and strive toward achieving the Healthy People 2030 targets for each cancer screening type.

## Supplementary Material

pkae113_Supplementary_Data

## Data Availability

The data for this study are existing public use data from the Centers for Disease Control and Prevention. They are available for download here at https://www.cdc.gov/brfss/annual_data/annual_data.htm.
