# Supplementary material for: Rural-urban disparities and trends in cancer screening: an analysis of Behavioral Risk Factor Surveillance System data (2018-2022)
Source: JNCI Cancer Spectr. 2024 Nov 9;8(6):pkae113. doi: 10.1093/jncics/pkae113 (PMC11671142; doi:10.1093/jncics/pkae113)
Supplement: pkae113_Supplementary_Data [file pkae113_supplementary_data.docx]

SAS Code For Data Cleaning and Weighted Screening Prevalence Estimates

**Creation of up to date cervical cancer screening variable**;

**Data** LLCP2018_new;

SET LLCP2018;

YEAR="2018";

IF _STATE in (**66**,**72**) then delete;

**CERVICAL CANCER SCREENING**;

EligibleCerv =(HADHYST2=**2** AND SEX1 = **2** AND _AGE80 in (**21**:**65**));

PAP_UPDATE= (HADPAP2=**1** AND LASTPAP2 in (**1**:**3**));

HPV_UPDATE= (HPVTEST=**1** AND HPLSTTST in (**1**:**4**));

Cerv_UPDATE= (PAP_UPDATE OR HPV_UPDATE);

**run**;

**Data** LLCP2020_new;

SET LLCP2020;

YEAR="2020";

IF _STATE in (**66**,**72**) then delete;

**CERVICAL CANCER SCREENING**;

EligibleCerv =(HADHYST2=**2** AND SEXVAR = **2** AND _AGE80 in (**21**:**65**));

PAP_UPDATE= (HADPAP2=**1** AND LASTPAP2 in (**1**:**3**));

HPV_UPDATE= (HPVTEST=**1** AND HPLSTTST in (**1**:**4**));

Cerv_UPDATE= (PAP_UPDATE OR HPV_UPDATE);

**run**;

**Data** LLCP2022_new;

SET LLCP2022;

YEAR="2022";

IF _STATE in (**66**,**72**) then delete;

EligibleCerv =(HADHYST2=**2** AND SEXVAR = **2** AND _AGE80 in (**21**:**65**));

*AnyScreen= (CERVSCRN=1 AND CRVCLCNC in (1:4));

PAP_UPDATE = (CRVCLPAP=**1** AND CRVCLCNC in (**1**:**3**));

HPV_UPDATE = (CRVCLHPV=**1** AND CRVCLCNC in (**1**:**4**));

Cerv_UPDATE= (PAP_UPDATE OR HPV_UPDATE);

**run**;

**Creation of combined dataset for BRFSS Survey Cycles and collapsed covariates**;

**data** BRFSS.CombinedBRFSS2018_2022;

set LLCP2018_new LLCP2020_new LLCP2022_new;

IF INCOME2 in (**1**:**4**) then Income=**1**; **lESS THAN 25K**;

IF INCOME2 in (**5**:**6**) then Income=**2**; ** 25-50k**;

IF INCOME2=**7** then Income=**3**; ** 50-75K**;

IF INCOME2=**8** then Income=**4**; ** >75K**;

IF INCOME2 in (**77**:**99**) then Income=**.**;

IF INCOME3 in (**1**:**4**) then Income=**1**; **LESS THAN 25K**;

IF INCOME3 in (**5**:**6**) then Income=**2**; **25-50**;

IF INCOME3=**7** then Income=**3**; ** 50-75K**;

IF INCOME3 IN (**8**:**11**) then Income=**4**; **>75k**;

IF INCOME3 in (**77**:**99**) then Income=**.**;

IF MEDCOST=**1** THEN MEDICALCOST=**1**; **YES, CANNOT SEE DOCTOR BECAUSE COST**;

IF MEDCOST=**2** THEN MEDICALCOST=**2**; **NO, COST NOT AN ISSUE**;

IF MEDCOST in (**7**:**9**) THEN MEDICALCOST=**.**; **MISSING**;

IF MEDCOST1=**1** THEN MEDICALCOST=**1**; **YES, CANNOT SEE DOCTOR BECAUSE COST**;

IF MEDCOST1=**2** THEN MEDICALCOST=**2**; **NO, COST NOT AN ISSUE**;

IF MEDCOST1 in (**7**:**9**) THEN MEDICALCOST=**.**; **MISSING**;

IF HLTHPLN1=**1** then INSURANCE=**1**; **HAVE SOME FORM OF INSURANCE**;

IF HLTHPLN1 in (**2**:**7**) then INSURANCE=**0**;**DO NOT HAVE SOME FORM OF INSURANCE**;

IF HLTHPLN1=**9** then INSURANCE=**.**;

IF PRIMINSR IN (**1**:**10**) then INSURANCE=**1**;**HAVE SOME FORM OF INSURANCE**;

IF PRIMINSR =**88** or PRIMINSR=**77** then INSURANCE=**0**;**DO NOT HAVE SOME FORM OF INSURANCE**;

IF PRIMINSR=**99** then INSURANCE=**.**;

IF EDUCA in (**1**:**3**) then EDUSTATUS=**1**; **Less than HS**;

IF EDUCA=**4** then EDUSTATUS=**2**; **HS grad**;

IF EDUCA=**5** then EDUSTATUS=**3**; **Some college**;

IF EDUCA=**6** then EDUSTATUS=**4**; **College Grad**;

IF EDUCA=**9** then EDUSTATUS=**.**; **College Grad**;

IF _CRCREC2=**1** then CRCREC2=**1**;

IF _CRCREC2=**2** then CRCREC2=**2**;

IF _CRCREC2=**3** then CRCREC2=**2**;

IF _CRCREC1=**1**then CRCREC1=**1**;

IF _CRCREC1=**2** then CRCREC1=**2**;

IF _CRCREC1=**3** then CRCREC1=**2**;

BreastScreened= coalesce(_MAM5022, _MAM5023);

CRCScreened= coalesce (_CRCREC, CRCREC1, CRCREC2);

**run**;

**Prevalence of screening estimates overall and by rural/urban status**;

**CERVICAL CANCER SCREENING**;

**PROC** **SURVEYFREQ** DATA=BRFSS.CombinedBRFSS2018_2022;

BY YEAR;

WHERE EligibleCerv=**1**;

TABLE Cerv_Update Cerv_Update*_URBSTAT / row col CL chisq;

WEIGHT _LLCPWT;

STRATA _STSTR;

CLUSTER _PSU;

**run**;

**PROC** **SURVEYFREQ** DATA=BRFSS.CombinedBRFSS2018_2022;

BY YEAR;

TABLE BreastScreened CRCscreened CRCscreened*_URBSTAT BreastScreened*_URBSTAT / row col CL chisq;

WEIGHT _LLCPWT;

STRATA _STSTR;

CLUSTER _PSU;

**run**;
